# Identification of key genes and functional enrichment analysis of liver fibrosis in nonalcoholic fatty liver disease through weighted gene co-expression network analysis

**DOI:** 10.5808/gi.23051

**Published:** 2023-12-29

**Authors:** Yue Hu, Jun Zhou

**Affiliations:** Shenzhen InnoStar Institute of Biomedical Safety Evaluation and Research Co., Ltd., Shenzhen,518000, China

**Keywords:** functional enrichment analysis, gene set enrichment analysis, hub genes, nonalcoholic fatty liver disease, liver fibrosis, weighted gene co-expression network analysis

## Abstract

Nonalcoholic fatty liver disease (NAFLD) is a common type of chronic liver disease, with severity levels ranging from nonalcoholic fatty liver to nonalcoholic steatohepatitis (NASH). The extent of liver fibrosis indicates the severity of NASH and the risk of liver cancer. However, the mechanism underlying NASH development, which is important for early screening and intervention, remains unclear. Weighted gene co-expression network analysis (WGCNA) is a useful method for identifying hub genes and screening specific targets for diseases. In this study, we utilized an mRNA dataset of the liver tissues of patients with NASH and conducted WGCNA for various stages of liver fibrosis. Subsequently, we employed two additional mRNA datasets for validation purposes. Gene set enrichment analysis (GSEA) was conducted to analyze gene function enrichment. Through WGCNA and subsequent analyses, complemented by validation using two additional datasets, we identified five genes (*BICC1*, *C7*, *EFEMP1*, *LUM*, and *STMN2*) as hub genes. GSEA analysis indicated that gene sets associated with liver metabolism and cholesterol homeostasis were uniformly downregulated. *BICC1*, *C7*, *EFEMP1*, *LUM*, and *STMN2* were identified as hub genes of NASH, and were all related to liver metabolism, NAFLD, NASH, and related diseases. These hub genes might serve as potential targets for the early screening and treatment of NASH.

## Introduction

Nonalcoholic fatty liver disease (NAFLD) is a prevalent form of chronic liver disease that contributes to metabolic disorders and associated health conditions. In recent years, the incidence of NAFLD has risen, surpassing viral hepatitis as the leading chronic liver disease worldwide. NAFLD severity varies from the milder nonalcoholic fatty liver (NAFL) to the more serious nonalcoholic steatohepatitis (NASH) [1,2]. NASH is characterized by hepatic steatosis accompanied by lobular inflammation and cell death, potentially progressing to fibrosis [3,4], cirrhosis, and even liver cancer. Notably, the degree of liver fibrosis is directly linked to the increased risk of liver cancer [5]. Consequently, evaluating the stage of liver fibrosis is crucial for the timely intervention in NASH. Liver fibrosis is classified into five stages: nonfibrotic (F0), mild fibrosis (F1), moderate fibrosis (F2), severe fibrosis (F3), and cirrhosis (F4) [6].

The occurrence and development of NAFLD and NASH are influenced by a range of factors [7,8], including genetic predisposition to obesity, epigenetic modifications, metabolic and signaling pathways in hepatocytes, and cellular interactions within the liver and adipose tissue [9]. Consequently, there is a need to develop an early noninvasive diagnostic system and an early warning system for disease risk. These would facilitate the identification of susceptibility genes for NASH, thereby assisting in the investigation of its pathogenesis and the development of potential treatments.

Weighted gene co-expression network analysis (WGCNA) is a method used to analyze gene expression patterns across multiple samples [10]. WGCNA clusters genes with similar expression profiles and examines the relationship between these clusters, known as modules, and specific traits or phenotypes. Additionally, it utilizes these modules and associated phenotypic data to identify central, or hub, genes within the modules. Consequently, WGCNA has become a widely employed tool in studies of phenotypic traits and gene association analyses, aiding in the identification of molecular markers or potential therapeutic targets in complex diseases [11,12].

We hypothesized that certain gene modules or hub genes play a significant role in the progression of liver fibrosis. For this study, we selected three sets of NASH data from the National Center for Biotechnology Information (NCBI). We performed WGCNA on the transcriptome data and corresponding liver fibrosis data to investigate the underlying mechanisms of NASH. Furthermore, we proposed that these hub genes may represent viable therapeutic targets for NASH.

## Methods

### Data collection and processing

The mRNA expression data utilized in our study, specifically from datasets GSE49541, GSE48452, and GSE167523, were retrieved from the Gene Expression Omnibus database at NCBI [13]. The GSE49541 dataset comprises expression data obtained through array profiling, focusing on NAFLD in 72 patients. This group included 40 individuals with mild NAFLD (fibrosis stages 0–1) and 32 with advanced NAFLD (fibrosis stages 3–4). The objective was to delineate liver gene expression patterns that differentiate mild from advanced NAFLD and to establish a gene expression profile linked to advanced NAFLD. The GSE48452 dataset also involved expression profiling by array, encompassing 73 human liver samples categorized into four groups: control (C; n = 14), healthy obese (H; n = 27), steatosis (S; n = 14), and NASH (n = 18). Data from the NASH group (N; n = 18), which included four samples with fibrosis stages 3–4 and 14 with fibrosis stages 0-1, were specifically selected for differential gene expression (DEG) analysis.

The GSE167523 dataset originates from global RNA sequencing of snap-frozen liver tissue obtained from 98 patients, comprising 48 with mild NAFLD and 50 with NASH, all of whom had biopsy-proven NAFLD. This data was generated using high-throughput sequencing.

The GSE49541 dataset was utilized to construct a co-expression network and identify hub genes associated with liver fibrosis in NAFLD. This microarray data provided a gene expression profile of the liver from 32 patients with advanced NAFLD (fibrosis stages 3–4) and 40 patients with mild NAFLD (fibrosis stages 0–1). The GSE49541 dataset underwent independent normalization using robust multiarray analysis (RMA) [14] at the NCBI, followed by log2 transformation and quantile normalization. To mitigate batch effects, ComBat was applied to the normalized combined dataset.

### Identification of DEGs

DEGs from GSE49541 between patients with advanced and mild NAFLD were identified in the expression data using the "limma" package in R via GEO2R on the NCBI platform [15]. The significance analysis of microarrays method was employed to detect genes with significant expression changes, applying a false discovery rate of <0.05 and an absolute log2 fold change of ≥0.5. DEGs from GSE48452 and GSE167523 were analyzed in the same manner as described above.

### Functional enrichment analysis

Gene ontology (GO) enrichment and Kyoto Encyclopedia of Genes and Genomes (KEGG) analyses of DEGs in various modules were conducted online via the GEne SeT AnaLysis Toolkit (http://www.webgestalt.org/) [16]. We established an adjusted p-value of <0.05 as the threshold for significance. All findings were visually represented using the "ggplot2" package in R [17].

### WGCNA and co-expression network construction

The R package "WGCNA" [10] was utilized to construct a co-expression network of DEGs using the GSE49541 microarray dataset. A soft-thresholding power of 22, an R^2^ cut-off value of 0.85, and a minimum module size of 25 genes were selected for the analysis. The "Bicor" correlation algorithm and a "signed" network type were employed in the network construction.

### Identification of hub genes

In the module-trait correlation analysis, hub genes exhibiting a Pearson correlation value greater than 0.4 and a p-value less than 0.0005 were identified as candidates with a significant correlation with the level of liver fibrosis. Subsequently, these genes were cross-referenced with DEGs from two other datasets (GSE48452 and GSE167523) to select common DEGs that demonstrated the same significant alterations.

### Gene set enrichment analysis

To further investigate the potential roles of the identified hub genes in NAFL fibrosis, gene set enrichment analysis (GSEA) was carried out for each hub gene individually [18]. The "clusterProfiler" R package was employed to perform the GSEA [19]. The reference gene set used was h.all.v7.4.entrez.gmt from the Molecular Signatures Database (MSigDB) [20], and an adjusted p-value of less than 0.05 was set as the filter condition.

### Statistical analysis

The statistical significance of differences between the two groups was assessed using either a nonparametric test or the t-test, depending on the characteristics of the data distribution. All analyses were performed with R software version 4.1.0 (R Foundation for Statistical Computing, Vienna, Austria). p-values less than 0.05 were deemed to indicate statistical significance.

## Results

### DEGs between advanced NAFLD and mild NAFLD

**Fig. 1.**
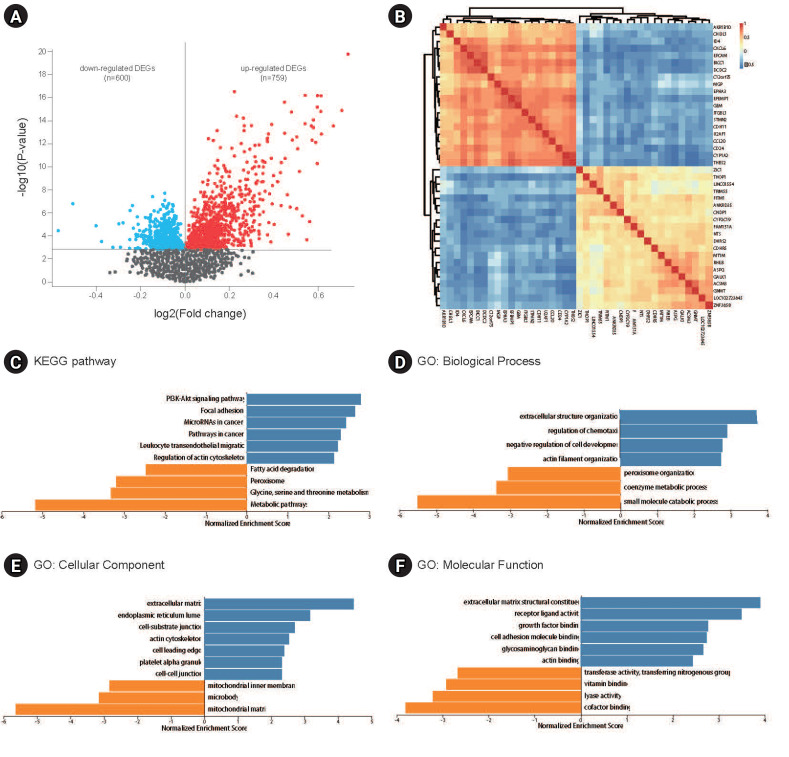
Differentially expressed gene (DEG) analysis and gene ontology (GO) and Kyoto Encyclopedia of Genes and Genomes (KEGG) analysis. (A) Volcano plot of DEGs in GSE49541. (B) Heatmap of the expression levels of the top 20 upregulated and top 20 downregulated DEGs. (C) KEGG analysis of downregulated and upregulated DEGs. (D–F) GO analysis of downregulated and upregulated DEGs.

A total of 1,359 DEGs, comprising 600 downregulated and 759 upregulated DEGs in GSE49541, were identified by comparing the transcriptomes of liver tissues from patients with advanced and mild NAFLD (Fig. 1A). These DEGs were subsequently utilized for WGCNA and the construction of a co-expression network. The correlations between the top 20 upregulated and the top 20 downregulated DEGs are depicted in Fig. 1B. KEGG pathway analysis showed that the upregulated DEGs were predominantly enriched in pathways such as phosphoinositide 3-kinase–Akt signaling, focal adhesion, microRNAs in cancer, cancer pathways, leukocyte transendothelial migration, and actin cytoskeleton regulation. In contrast, downregulated genes were enriched in pathways including fatty acid degradation, peroxisome, and the metabolism of glycine, serine, and threonine, as well as other metabolic pathways (Fig. 1C). GO analysis indicated that these DEGs are implicated in biological processes such as extracellular structure organization, regulation of chemotaxis, small molecule catabolic processes, and cellular components including the extracellular matrix, endoplasmic reticulum lumen, and mitochondrial matrix. They are also involved in molecular functions like structural constituents of the extracellular matrix, receptor ligand activity, and cofactor binding (Fig. 1D).

### WGCNA analysis and co-expression network construction

**Fig. 2.**
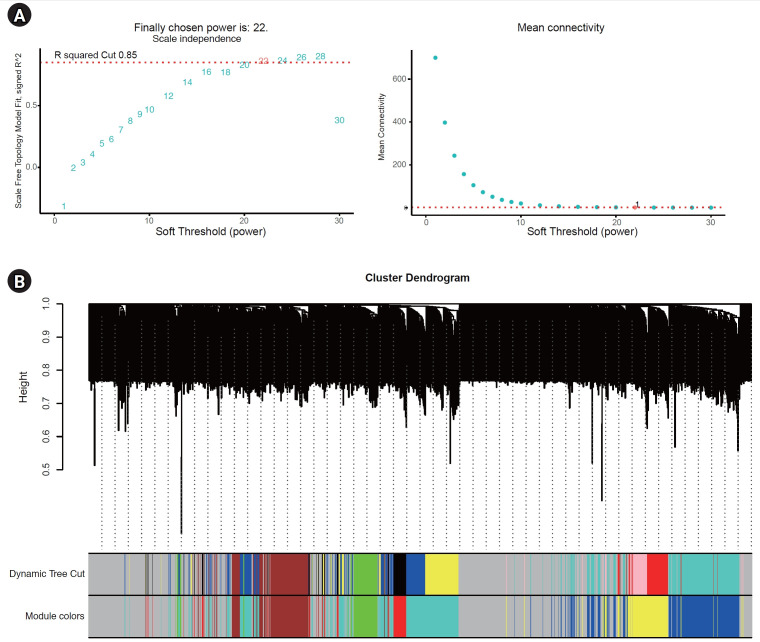
Co-expression module analysis. (A) The relationship between the scale-free fit index and various soft-thresholding powers and between the mean connectivity and various soft-thresholding powers. (B) Clustering dendrogram of genes; various colors represent different modules.

We selected a correlation coefficient threshold of 0.85, and the soft-thresholding power was determined to be 22 (Fig. 2A). Seven co-expression modules were identified using WGCNA (Fig. 2B). While the gray module contained the largest number of genes, it did not include any genes that were significantly correlated. Consequently, the turquoise module contained the majority of significantly correlated genes, with the blue, brown, and yellow modules following in that order (Fig. 2B).

### Module-trait correlations in liver fibrosis and the identification of hub genes

**Fig. 3.**
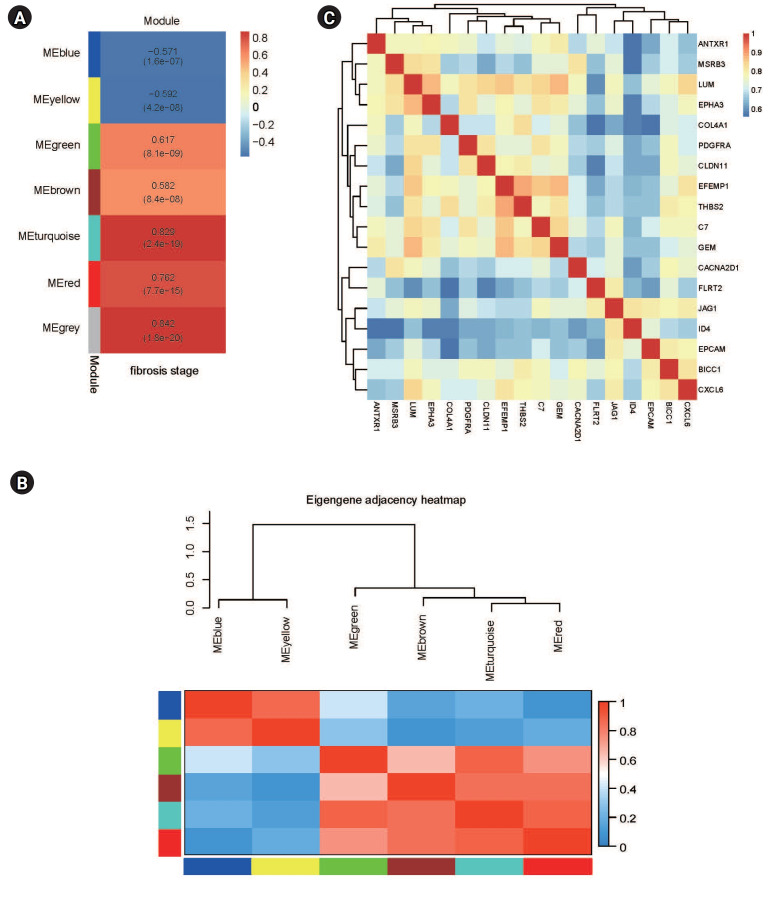
Important module analysis. (A) The relationships between the liver fibrosis trait and seven modules. (B) Eigengene adjacency heatmap of differentially expressed gene expression levels in six modules. (C) Heatmap of the relationships among genes with high correlation values (Pearson correlation value > 0.7, p < 0.05) for liver fibrosis.

The analysis revealed that seven distinct modules were associated with varying degrees of NAFL fibrosis (Fig. 3A). The DEGs within the turquoise module exhibited the strongest positive correlation with the most advanced stage of liver fibrosis, whereas the DEGs in the yellow module demonstrated the most pronounced negative correlation. The DEGs in the turquoise, red, brown, and green modules showed increased expression, in contrast to the downregulated DEGs in the blue and yellow modules. The module eigengene adjacency heatmap displayed the gene expression patterns across these modules (Fig. 3B). Correlation analysis, as detailed in Table S1 and derived from WGCNA, revealed that genes with high correlation values (Pearson correlation value > 0.7, p < 0.05) in the context of liver fibrosis also exhibited a strong interrelationship (Fig. 3C). Consequently, these genes were identified as potential hub gene candidates.

### Validation and efficacy evaluation of hub genes

**Fig. 4.**
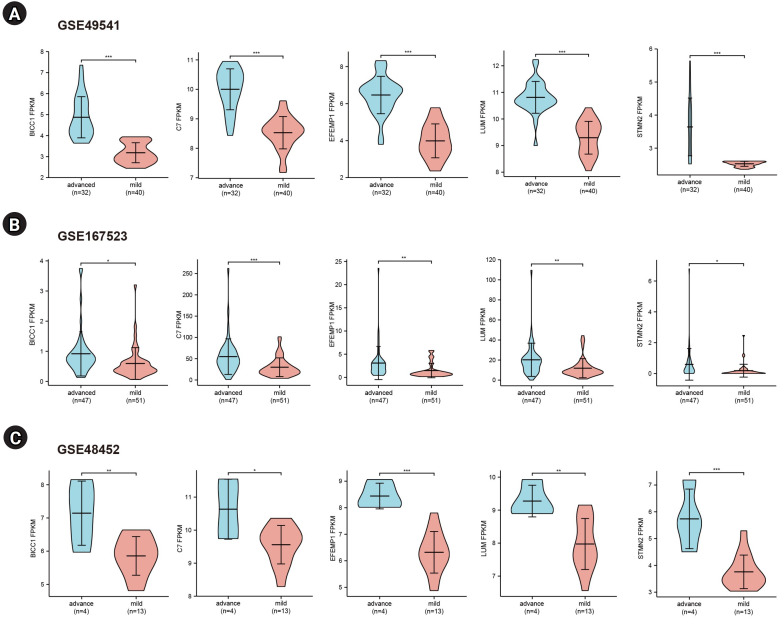
Gene expression levels of the five key genes in three mRNA datasets.

To further validate the hub genes, we selected two additional transcriptome datasets (GSE48452 and GSE167523) from liver tissues of patients with advanced and mild NAFLD. Upon comparison with the GSE49541 dataset, we identified five key DEGs (*BICC1*, *C7*, *EFEMP1*, *LUM*, and *STMN2*) that exhibited consistent and significant upregulation in both datasets (Fig. 4). Moreover, we conducted receiver operating characteristic curve analysis and calculated the area under the curve (AUC) to differentiate between advanced fibrosis (stage 3–4) and mild fibrosis (stage 0–1). The analysis revealed that the AUCs for these five genes were all greater than 0.7 across the datasets GSE49541 (Supplementary Fig. 1A), GSE167523 (Supplementary Fig. 1B), and GSE48452 (Supplementary Fig. 1C).

### Gene set enrichment analysis

**Fig. 5.**
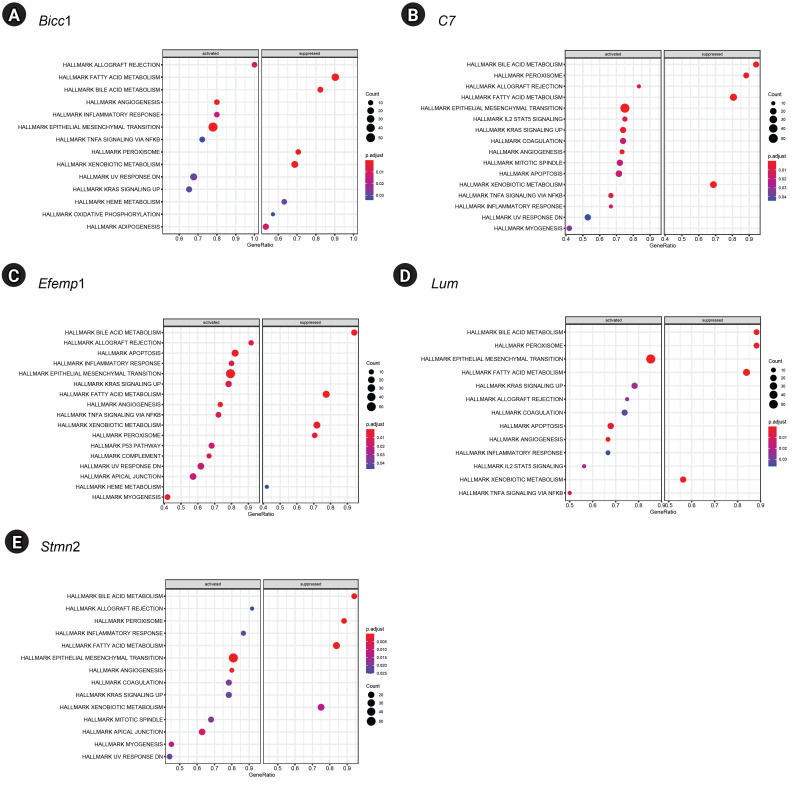
Gene set enrichment analysis results for five key genes. (A) *Bicc1*. (B) *C7*. (C) *Efemp1*. (D) *Lum*. (E) *Stmn2*.

**Fig. 6.**
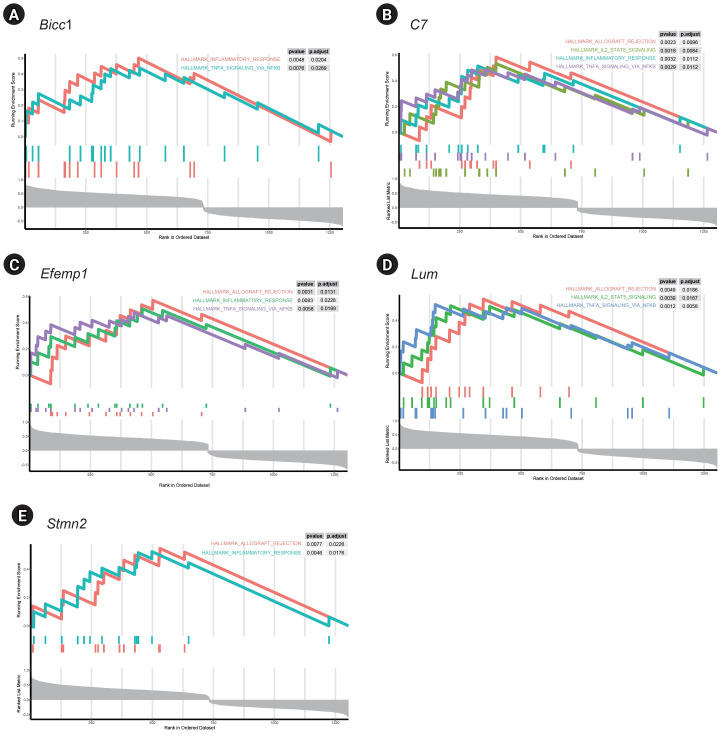
Pathway analysis of gene sets related to immunity in five key genes. (A) *Bicc1*. (B) *C7*. (C) *Efemp1*. (D) *Lum* (E) *Stmn2*.

GSEA of single genes revealed that the gene sets were enriched in the samples with *BICC1* (Fig. 5A), *C7* (Fig. 5B), *EFEMP1* (Fig. 5C), *LUM* (Fig. 5D), and *STMN2* (Fig. 5E). While these gene sets showed high expression, others were suppressed, including those involved in fatty acid metabolism and bile acid metabolism—critical pathways in liver metabolism and cholesterol homeostasis. We focused on gene sets associated with immunity for further analysis. We found that two gene sets, specifically those related to the inflammatory response and tumor necrosis factor (TNF)-α signaling via NF-κB, were enriched in samples with elevated expression of *BICC1*, *C7*, and *EFEMP1*. Additionally, gene sets associated with allograft rejection were also enriched in samples with *C7* and *EFEMP1*, while those related to interleukin (IL)-2-STAT5 signaling were enriched in samples with *C7* (Fig. 6A-C). Similarly, gene sets linked to allograft rejection, IL2-STAT5 signaling, and TNFα signaling via NF-κB were enriched in samples with *LUM* (Fig. 6D), and those related to allograft rejection and inflammatory response were enriched in samples with *STMN2* (Fig. 6E).

## Discussion

NAFLD is the most common chronic liver disease worldwide, encompassing a spectrum of pathological processes from benign hepatic steatosis to NASH, cirrhosis, and potentially hepatocellular carcinoma [21]. The progression from simple hepatic steatosis to NASH represents a critical juncture in the evolution of severe liver disease. Patients with NASH face a substantially increased risk of liver fibrosis and end-stage liver disease compared to those with simple fatty liver disease [22]. Consequently, pinpointing genes that predispose individuals to NASH is instrumental for understanding its pathogenesis and for the development of targeted therapies.

Recent studies have shown that it is necessary to build gene co-expression networks within the scope of exploratory research. These networks are instrumental in identifying key modules and genes associated with specific diseases. In our study, we employed WGCNA to examine NASH transcriptome data (GSE49541). We discovered that the turquoise module exhibited the most significant positive correlation with NASH and liver fibrosis, whereas the yellow module demonstrated the most significant negative correlation. To further pinpoint hub genes, we compared DEGs from two additional transcriptome datasets (GSE48452 and GSE167523). This comparison revealed five common genes (*BICC1*, *C7*, *EFEMP1*, *LUM*, and *STMN2*) that were consistently upregulated. The AUC values for these five hub genes were greater than 0.7 across the datasets, confirming the reliability of our analytical approach.

The functions of these five genes are all associated with liver metabolism, NAFLD, NASH, and related conditions. *LUM* is a novel essential factor in hepatic fibrosis and encodes an extracellular matrix proteoglycan [23]. It has also been identified as a central gene in the progression of fibrosis in patients with NAFLD [24]. *C7*, which encodes a serum glycoprotein involved in forming a membrane attack complex, has been suggested as a potential biomarker for advanced fibrosis in NAFLD through proteomic screening [25] and is implicated in the disease's mechanism [26]. *EFEMP1* is recognized as a transcriptomic signature in NASH [27]. *STMN2* has been profiled in early-stage liver fibrosis in patients with chronic hepatitis C virus infection [28], and its expression has been positively correlated with insulin resistance in NASH [29]. *BICC1* has been identified as a novel prognostic biomarker in gastric cancer, associated with immune infiltrates [30], and has also been suggested as a diagnostic marker for NAFLD [31]. GSEA of these five genes further confirmed their roles in liver metabolism. For instance, disruptions in bile acid metabolism can lead to cholestatic liver disease, dyslipidemia, fatty liver disease, cardiovascular disease, and diabetes [32].

## Supplementary Materials

Supplementary Fig. 1. The area under the curve (AUC) results, as obtained through receiver operating characteristic curve analysis. FPR, false positive rate; TPR, true positive rate.
