# Supplementary figures and images for: Identification of key genes and functional enrichment analysis of liver fibrosis in nonalcoholic fatty liver disease through weighted gene co-expression network analysis
Source: Genomics Inform. 2023 Dec 29;21(4):e45. doi: 10.5808/gi.23051 (PMC10788356; doi:10.5808/gi.23051)

**(A)**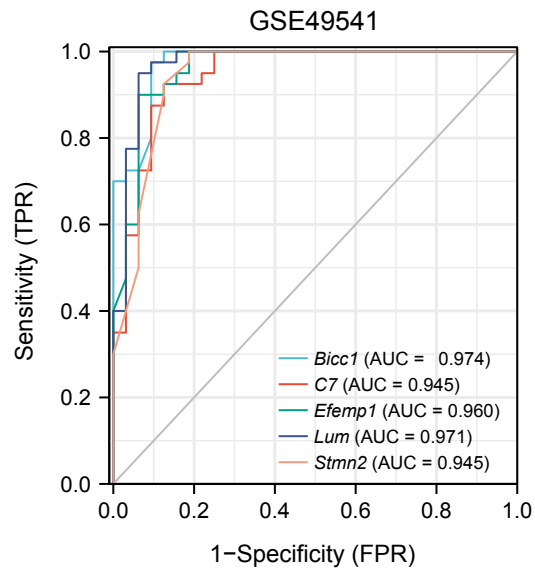**(B)**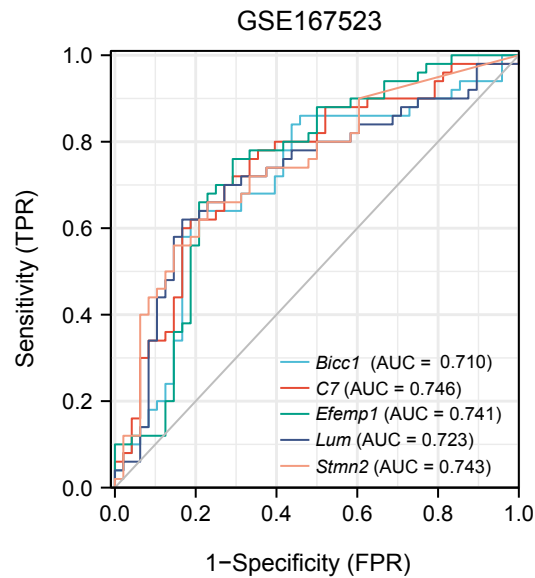**(C)**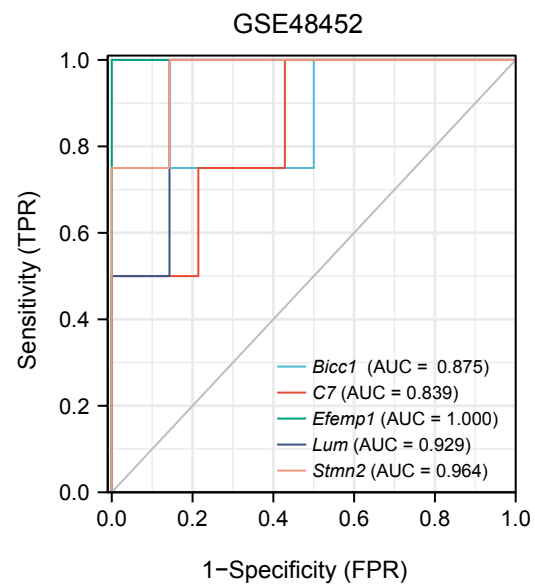

Supplement: Supplementary Fig. 1. — The area under the curve (AUC) results, as obtained through receiver operating characteristic curve analysis. FPR, false positive rate; TPR, true positive rate. [file gi-23051-Supplementary-Fig-1.pdf]
